# Characteristics and phylogenetic analysis of the complete chloroplast genome of *Paeonia japonica* (Paeoniaceae)

**DOI:** 10.1080/23802359.2020.1860718

**Published:** 2021-03-11

**Authors:** Seo-Young Lim, Ji-Hun Jang, Hyun-Ju Lee, Seong-Sik Park, Sun-Ra Kim, Kyeong-Min Lee, Ji-Kyung Kim, Ho Park, Ho-Kyung Jung

**Affiliations:** National Development Institute of Korean Medicine, Jangheung-gun, South Korea

**Keywords:** Chloroplast genome, *Paeonia japonica*, phylogenetic tree analysis

## Abstract

*Paeonia japonica*, distributed throughout Asia, is a traditional medicinal herb in Korea, with many potential beneficial effects including pain-relieving, anti-inflammatory, and anti-cancer activities. Despite its high pharmacological value, the genetic information on *Paeonia japonica* remains limited. In this study, the chloroplast genome of *P. japonica* was sequenced using next-generation sequencing (NGS) technology and genome and phylogeny were analyzed using multiple tools. The chloroplast genome of *P. japonica* was 152,731 bp in length with an inverted repeat region of 26,656 bp, including a large single-copy region of 84,389 bp and a small single copy region of 17,030 bp. The *P. japonica* chloroplast genome included 113 genes comprising 80 protein-coding genes, 27 tRNA, and 5 rRNA genes. Phylogenetic analysis indicated that *P. japonica* and *P. obovata* share a close evolutionary relationship.

*Paeonia japonica*, belonging to the family Paeoniaceae, is distributed throughout Asia and its roots have long been used for medicinal purposes in South Korea. *P. japonica* is effective for treating pain (Zhang et al. 2014) and gynecological diseases (Xu et al. 2019). It is also known to have anti-inflammatory (Wu et al. 2010), vascular expansion (Park et al. 2009), and anti-tumor effects (Tan et al. 2020). Despite its pharmacological importance, limited genetic information about *P. japonica* is available. Thus, in this study, we identified the sequence of the complete chloroplast genome sequence of *P. japonica* using next-generation sequencing techniques and then performed phylogenetic analysis.

Samples of *P. japonica* were collected from Goesan-gun (Chungcheongbuk-do, South Korea, 36°45'47.7"N 127°53'24.8"E) and Yangju-si (Gyeonggi-do, South Korea, 37°48'15.1"N 127°01'32.6"E). Voucher specimens (TKMII-23-2) were deposited to the Medicinal Crops Seed Supply Center of the National Institute for Korean Medicine Development. Chloroplast DNA was extracted using the DNeasy Plant Mini Kit (Qiagen, Germany) and DNA library was created using EBNext Ultra II DNA Library Prep Kit for Illumina (NEB). The generated library used TapeStation HSD5000 (Agilent) to analyze the condition and determine the suitability of NGS analysis. The prepared library sequenced using the Illumina HiSeq 2500 Platform with 151 bp paired-end reads at Genotech (Daejeon, Korea). About 3.25 Gb raw reads (9639.196 bp) were generated and assembled using NOVOPlasty v2.6.7 (Dierckxsens et al. 2017). Gene annotation was performed using GeSeq (Tillich et al. 2017) and CPGAVAS2 (Shi et al. 2019). When performing assembly and annotation, the Paeonia lactiflora chloroplast genome (GenBank accession: NC_040983.1) was used as a reference genome. The annotated chloroplast genome sequence was submitted to the NCBI GenBank database under the accession number MT821944. The circular genome annotation map was drawn using OGDRAW v1.3.1 (Greiner et al. 2019). Next, to determine the phylogenetic position of *P. japonica*, 19 complete chloroplast genomes belonging to the Paenoniaceae and 4 outgroup sequences were aligned using MAFFT v7 (Katoh and Standley 2013). A maximum likelihood phylogenetic tree was generated using MEGA X (Kumar et al. 2018) with 1000 bootstrap replicates.

The complete chloroplast genome of *P. japonica* was 152,731 bp in length and included a pair of inverted repeats (IRa and IRb) spanning 25,656 bp, separated by a large single copy region (LSC) of 84,389 bp and a small single copy region (SSC) of 17,030 bp. Overall, the GC content of the *P. japonica* chloroplast genome was 38.4%, and that of the LSC and SSC regions was 36.7% and 32.7%, respectively. In total, the *P. japonica* chloroplast genome included 113 genes. These included 80 protein-coding genes, 27 tRNA genes, and 5 rRNA genes. The maximum likelihood phylogenetic tree showed that *Paeonia* species were clustered together (Figure 1), and that *P. japonica* was closely related to *P. obovata*. This decoded chloroplast genome sequence of *P. japonica* will be useful for studying species conservation strategies and pharmacological efficacy in the future.

**Figure 1.**
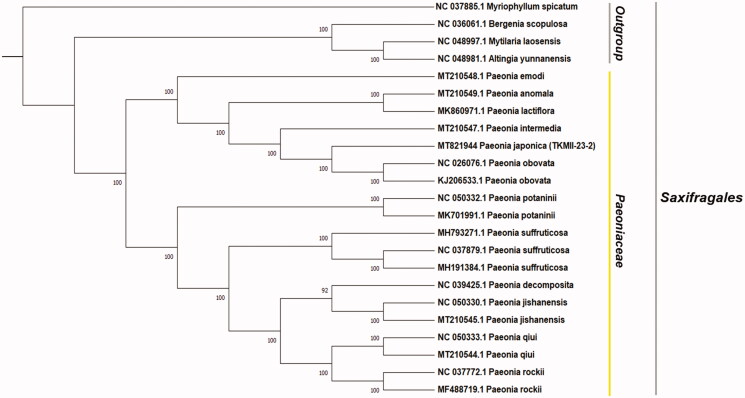
The ML phylogenetic tree constructed with total 23 chloroplast genome sequences. (Bootstrap replicates = 1000). All the sequences were downloaded from GenBank.

## Data Availability

The data generated in this study are publicly available at NCBI GenBank (https://www.ncbi.nlm.nih.gov/genbank/), under reference number MT821944.
